# Participation in mass dog vaccination campaigns in Tanzania: Benefits of community engagement

**DOI:** 10.3389/fpubh.2022.971967

**Published:** 2022-10-13

**Authors:** Christian Tetteh Duamor, Felix Lankester, Emmanuel Mpolya, Elaine A. Ferguson, Paul CD. Johnson, Sally Wyke, Sarah Cleaveland, Katie Hampson, Katharina Kreppel

**Affiliations:** ^1^Department of Global Health and Biomedical Sciences, School of Life Sciences and Bioengineering, Nelson Mandela African Institution of Science and Technology, Arusha, Tanzania; ^2^Environmental Health and Ecological Sciences Thematic Group, Ifakara Health Institute, Dar-es-Salaam, Tanzania; ^3^Boyd Orr Centre for Population and Ecosystem Health, Institute of Biodiversity, Animal Health and Comparative Medicine, College of Medical, Veterinary and Life Sciences, University of Glasgow, Glasgow, United Kingdom; ^4^Paul G. Allen School for Global Health, Washington State University, Pullman, WA, United States; ^5^Global Animal Health Tanzania, Arusha, Tanzania; ^6^School of Social and Political Sciences, School of Health and Wellbeing, College of Social Sciences, University of Glasgow, Glasgow, United Kingdom; ^7^Department of Public Health, Institute of Tropical Medicine, Antwerp, Belgium

**Keywords:** community, public, engagement, rabies, mass dog vaccination

## Abstract

**Background:**

Canine rabies causes about 59,000 human deaths each year globally but the disease can be eliminated by sustaining sufficient dog vaccination coverage over several consecutive years. A challenge to achieving high coverage is low participation of dog owners in vaccination campaigns. We explored whether and how previously identified contributory factors to low participation can be addressed through community engagement activities.

**Methods:**

We engaged communities in two wards in Tanzania on dog behavior and handling, safe ways of interacting with dogs, and their perceptions of dog vaccination. We shared and elicited information from them through village meetings, video screenings, posters and leaflets and involved the leadership of one of the wards in planning and implementing a dog vaccination exercise to explore the feasibility of their participation. We assessed the impact of engagement activities with household surveys, meeting reports, observations and focus group discussions. We used a generalized linear mixed-effects model to identify predictors of knowledge and perceptions and compared knowledge amongst respondents before and after engagement activities. Qualitative data was analyzed inductively to explore perceptions of dog handling and vaccination and feasibility, opportunities and barriers to community leadership participation in organizing mass dog vaccination.

**Main findings:**

Knowledge of dog behavior, dog handling, and safe ways of interacting with dogs was positively associated with age (*p* < 0.0001), dog ownership (*p* = 0.0203), training (*p* = 0.0010) and previous experience of a dog bite (*p* = 0.0002); and was negatively associated with being afraid of dogs (*p* = 0.0061) and participation in a recent dog vaccination campaign (*p* = 0.0077). Knowledge was low before and significantly improved after engagement activities. The majority (92%) of respondents believed dog vaccination has no negative effects on dogs. Respondents perceived lack of bonding with their dog as a limitation to the ability to restrain a dog for vaccination. The community performed most roles assigned to them in the dog vaccination exercise, but barriers such as lack of motivation for volunteering exist.

**Conclusion:**

Engaging communities regularly on dog vaccination can improve their knowledge of dog behavior and dog handling techniques, and may help improve owner participation in dog vaccination campaigns.

## Introduction

Rabies is a zoonotic, viral infection of the central nervous system of mammals that causes about 59,000 human deaths each year globally (1). In Tanzania, rabies-specific human deaths are estimated at 552 [394-731] per annum (2). The main sources of infection to humans are domestic dogs, with infection always resulting in death once clinical signs occur (3, 4). Sustaining vaccination coverage above 40% over several years can interrupt rabies virus transmission within the dog population and therefore prevent transmission to people (3, 5, 6). On this premise, the Tripartite, comprising the World Health Organization (WHO), the World Organization for Animal Health (WOAH) and the Food and Agriculture Organization (FAO), have developed a global target to eliminate human deaths from rabies by 2030. However, current mass dog vaccination campaign approaches often fail to achieve the vaccination coverage needed to sustain herd immunity throughout the year, partly because of low levels of dog owner participation (7–9). Studies of mass dog vaccination campaigns in Tanzania show coverage is usually below 50% (9–12).

Several factors have been shown to contribute to low levels of dog owner participation, including fear of dogs (13), poor knowledge of dog behavior (14), poor dog handling techniques (7, 13–16), lack of appreciation of dogs' welfare (17), and negative perceptions of the impact of vaccination on dogs (16). A process evaluation of community-based dog vaccination strategies in the Mara region of Tanzania reported some negative perceptions toward dog vaccination, such as vaccination causing a dog to die, develop skin rashes, become infertile, and be unable to hunt or guard property (39). These negative perceptions could stand in the way of dog owner participation in mass dog vaccination campaigns against rabies.

For many disease control programmes, community engagement approaches have been shown to deepen shared knowledge of disease control challenges between researchers and communities, resulting in better intervention design tailored to context and improved local participation in delivery and uptake (18–20). For dog rabies vaccination campaigns, experiences from Chad and Kenya have shown that community involvement also has the potential to reduce costs (21–23). Similarly, the successes of mass drug administration programmes for NTDs amenable to chemotherapy, including the community-directed treatment with ivermectin, was attributed to broad participation in design and delivery of the interventions (24), which empowered communities and enhanced acceptance (19).

We aimed to assess how previously identified contributory factors to low participation in dog vaccination in Tanzania could be addressed. We designed community engagement activities to share and elicit information on rabies from endemic communities. We adopted a range of approaches including village-level and school-based meetings, posters, leaflets, flipcharts and video screenings. We also held dog handling demonstrations and meetings with community leaders to plan a mass dog vaccination campaign.

The engagement activities were evaluated using a before-and-after design using mixed methods. Specifically, we explored (a) which population-related factors should be considered in tailoring sensitization interventions toward mass dog vaccination campaigns, (b) the effect of [participation in] engagement activities on knowledge of dog behavior/ body language interpretation, how to restrain dogs and safe ways to interact with dogs and perceptions of the impact of dog vaccination and (c) whether it was feasible to include community leaders in both planning and implementing mass dog vaccination campaigns.

## Methods

### Study site

**Figure 1:**
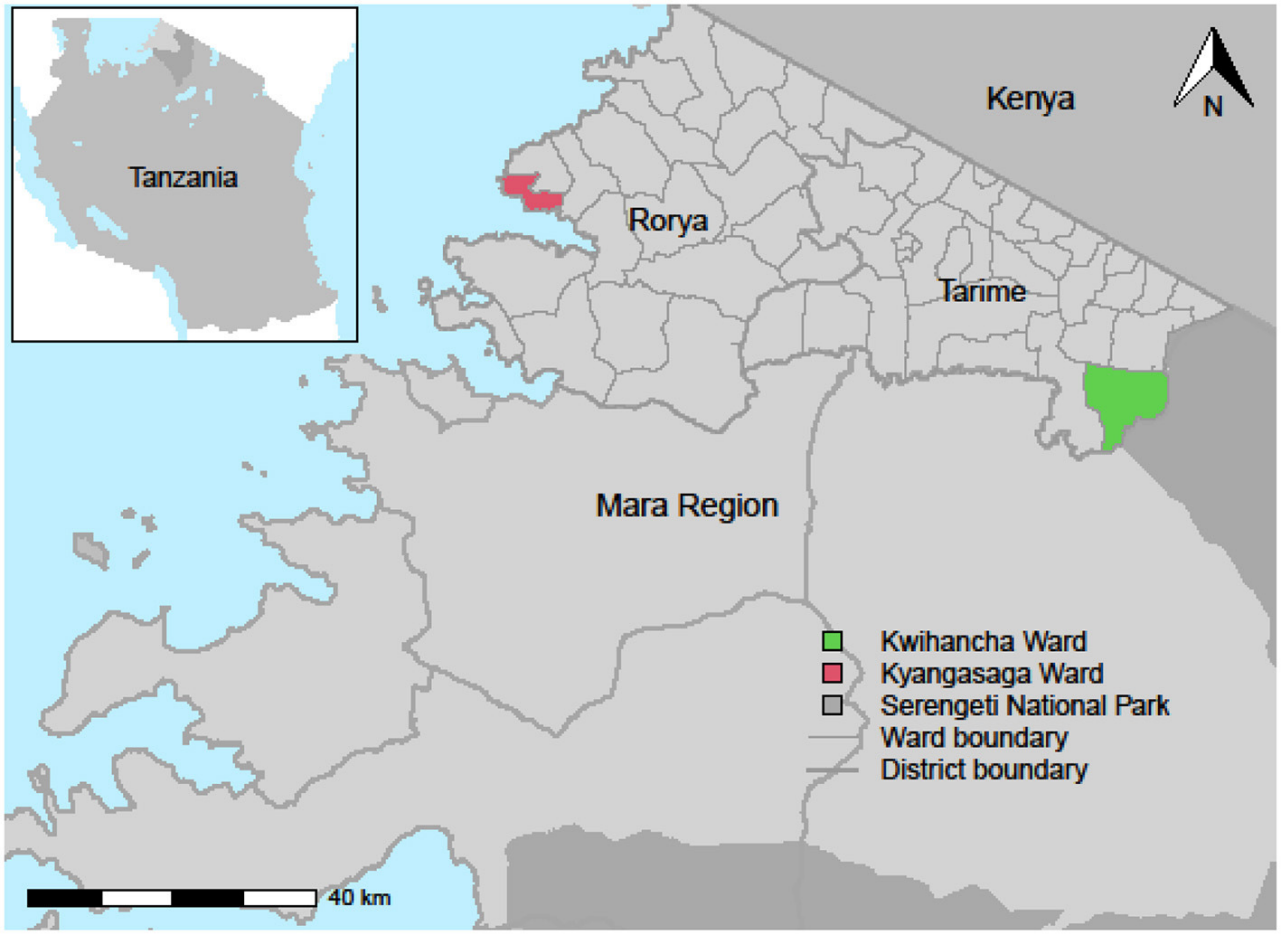
Map of Mara region in Tanzania showing locations of the two wards where the community and public engagement activities were conducted.

The community engagement activities were conducted in two wards, Kyangasaga (comprising Gabimori and Kyangasanga villages) and Kwihancha (comprising Gibaso, Karakatonga, and Nyabilongo villages) of Rorya and Tarime districts respectively. These districts are located in the Mara region of north-west Tanzania between Lake Victoria and Kenya, where dog rabies is endemic (Figure 1). At the time of this study, there was no regular mass dog vaccination in Kyangasaga ward, whereas dog vaccination had been conducted annually since 2003 in Kwihancha ward by the Serengeti Health Initiative (12).

### Study population

Both study wards comprised agropastoral communities, practicing mixed crop and livestock farming, although fishing is also a common practice in Kyangasaga (located on the bank of Lake Victoria). In agropastoral communities, dogs are kept primarily to guard livestock and farms and the number of dogs owned is typically associated with the number of livestock kept (25–27). In fishing communities, such as Kyangasaga, dogs can be considered a nuisance as they feed on sardines (which are dried in the open). Like other areas in Tanzania, teenagers and children mainly bring dogs to vaccination points (25, 26), and were an important target group for the community engagement.

### Study design

We conducted a before-and-after evaluation of the impact of community engagement on dog handling and vaccination and feasibility of involving village leadership in planning and implementing mass dog vaccination campaigns, using mixed methods.

#### Community engagement activities

##### Training and community engagement content development workshops

The engagement activities were conducted from January to June 2020. A team of eight members delivered the engagement activities. This team included: a research officer from the Nelson Mandela African Institution of Science and Technology (the first author - CTD) specializing in process evaluation of complex interventions, five field researchers including one livestock field officer, one police dog trainer, three community development experts with experience in field data collection, and two community informants. The team was trained on the concepts of community engagement by CTD and on how to use Open Data Kit (ODK) to collect data by a data scientist from the Ifakara Health Institute during a 5-day workshop. The concepts of community engagement on which the team was trained were: community engagement as a process co-authored with communities, effective community entry, facilitation of group-based participatory processes, community ownership of solutions to local problems (rabies), community empowerment through partnerships and ownerships, and the difference between community engagement and sensitization/ education. The team then worked together and developed the content of the engagement activities including posters, leaflets, flipcharts, videos and data collection tools in ODK. The engagement materials were developed with reference to the literature, online videos, text, images and international guides on recommended dog handling practices (Supplementary files 5A–E).

#### Implementation of community engagement

Four introduction meetings were held, two each with leaders of the two targeted wards. At the first meetings the purpose and activities of the community engagement events were discussed and support was sought for the engagement activities. This was followed by another meeting between the research team and leaders of the two wards to schedule the activities for their villages.

The activities carried out included: (i) sub-village level/ school-based forums on dog behavior, dog handling and dog vaccination, where the research team solicited the views of the community members and school children, and shared information using flip charts and short videos; (ii) dog handling demonstrations by owners and vaccinators; and (iii) distribution of posters and leaflets (on dog behavior, dog handling, rabies and dog vaccination) to households included in the baseline survey across the two wards. We expected engagement with the materials and attendance at a community forum to increase knowledge about dog behavior/ body language interpretation, how to restrain dogs and safe ways to interact with dogs, with an eventual increase in participation in vaccination campaigns.

#### Involvement of village leadership in vaccination campaigns

The research team, three members from Rorya district veterinary office, and 13 leaders of Kyangasaga wards (where dog vaccination campaigns had not been happening at the time of engagement) had a community-based meeting, lasting three and a half hours to plan a community-led mass dog vaccination campaign. The meeting had three agenda items: first, participants discussed the effects of rabies in their communities; second, participants identified and discussed key activities/ steps in organizing mass dog vaccination campaigns and third, participants identified key stakeholders in organizing mass dog vaccination and assigned roles to these stakeholders for the planned mass dog vaccination campaigns. This meeting was followed up with a community-led mass dog vaccination exercise after 3 months to demonstrate the actual performance of assigned roles. An observation was carried out on the vaccination exercise to identify factors that facilitated or impeded involvement of community leadership in the vaccination activities.

All activities were conducted in Kiswahili, the local language, and data were later translated into English.

### Engagement activities and their reach

Seven sub-village-level, plus three school-based (two secondary and one primary) meetings, two dog handling demonstration sessions and four meetings with village leadership were held. Altogether, 2,903 people participated in the engagement activities, including: 24 community leaders, 16 teachers and 729 students/ pupils. Eight hundred and twenty-six (826) people were reached with 2,000 posters and 1,000 leaflets in 375 households. Four hundred and seventy-eight and 805 community members were reached through sub-village level forums and video screenings respectively. Nine people who were dog handlers during vaccination campaigns and 16 dog owners participated in the dog handling demonstrations. Further details on participation in the various engagement activities are provided in Supplementary file 4.

## Evaluation

### Effect of community engagement activities

#### Household survey

##### Sampling

The effect of engagement activities was evaluated using before and after household surveys. The survey was piloted with 50 respondents in a nearby ward with a similar demographic profile.

We sampled households in each of the two wards by starting from the house of the sub-village chairperson and selecting every other house while moving east to west or vice versa across the settlement. In total, 375 households were selected from the two wards (*n* = 165 in Kyangasaga and *n* = 210 in Kwihancha – the bigger ward). All villages in each ward were targeted, while sub-villages were conveniently selected on the basis of accessibility to people. Following the information and consenting process at household level with the “head of household”, two to three respondents aged 14 years and above were interviewed per household, using a structured questionnaire designed in ODK. We interviewed *n* = 728 people from 375 households in the before and after surveys.

#### Measurements

##### Baseline

Data was collected on the demographic profile of participants, dog ownership, participants' knowledge of dog behavior, of dog handling and of safe ways of interacting with dogs, using a structured questionnaire with participant-self-rated and interviewer-rated responses.

Knowledge was measured in three areas:

(i) **Dog behavior** – assessed through ability to correctly interpret dog body language with 21 questions (accompanied with pictures showing dogs behaving in different ways). The interviewers then compared their answers to recommended interpretations.
(ii) **Dog handling** – assessed through ability to restrain at home, how to calm down a dog and recommended ways of holding big and small dogs during vaccination. Participants were asked to describe these activities, the interviewers then compared their answers to recommended practice and rated their answers as “not correct”, “partially correct” or “correct”.
(iii) **Safe ways of interacting with dogs** – assessed through knowledge of how to avoid dog attacks (11 questions) and how to limit injury in case of attack (five questions). Participants were asked to enumerate and explain ways to avoid dog attacks or limit injury when attacked, the interviewers then compared their responses to recommended steps. All questions are included in Supplementary files 1, 2.

To score answers in measuring knowledge of dog behavior and safe ways of interacting with dogs, a binary scale of zero (for incorrect answer) and one (for correct answer) was applied (28, 29). Correct answers were defined as one that conforms with what is generally recommended by expert literature (Supplementary files 5A–E). The interviewers were trained on these and had the information to hand for reference where necessary.

##### Follow up survey

Households were revisited 3 months after the baseline survey and engagement activities and the questionnaire repeated with respondents from the same households (*n* = 728 respondents). Additionally, respondents were asked through which medium of information (poster, leaflet, engagement activity) they received information on dog behavior and handling.

#### Vaccination coverage

Sub-village chairpersons went house-to-house to count the number of dogs in each household before the start of the vaccination exercise. The number of dogs vaccinated in each sub-village was recorded in a register. Vaccination coverage was expressed as percentage of dogs in the sub-village that were vaccinated.

#### Focus group discussions

Perceptions of the impact of dog vaccination, knowledge of dog behavior, knowledge of dog handling and knowledge of safe ways of interacting with dogs were further explored through focus group discussions (FGD). Four FGD (two per ward) were held after engagement activities, with participants purposively selected to ensure there was equal gender and community representation. Each FGD included nine people, and was conducted separately for young people (below 20 years of age) and adults of both genders. Discussions were conducted in Kiswahili by members of the research team who were experienced interviewers, using topic guides (Supplementary file 3). Discussions lasted about 40 min each and were recorded with an Olympus VN-541PC voice recorder. Informed consent was obtained from all respondents or their guardians.

#### Observations

We conducted participant observation of all engagement sessions, recorded using field notes written by CTD to consider further insights into societal underpinnings of perception of and participation in dog vaccination, and dog handling.

#### Involvement of village leadership in vaccination campaigns

We used participant observation of all meetings and activities together with formal reports on meetings to assess the feasibility of involving community leadership in mass dog vaccination. The performance of roles assigned to the key stakeholder groups and factors that influenced the involvement of community leadership were observed and documented during the vaccination exercise. These observations were conducted by CTD and recorded using a structured (qualitative) proforma (Supplementary file 6).

## Data analysis

### Quantitative data

#### Predictors of “knowledge” level of dog behavior, handling and safe ways to interact with dogs

To assess which population-related factors should be considered in tailoring sensitization interventions, we used a binomial, generalized linear mixed-effects model (fitted using the glmmTMB package (30)) to identify which of these factors are associated with the “knowledge” level score at baseline, before being exposed to engagement activities. The variables “ward”, “village” and “household” (nested within village) were included as random effects. The variable “knowledge” was the response variable and is an unweighted aggregation of the individuals” binary scores determined from three variables: (i) their knowledge of dog behavior (ability to correctly interpret dog body language), (ii) knowledge of dog handling (ability to restrain a dog at home, how to calm a dog down and recommended ways of holding big and small dogs during vaccination) and (iii) knowledge of safe ways of interacting with dogs (how to avoid dog attacks and how to limit injury in case of attack). Out of 14 explanatory variables originally considered, two were dropped due to strong correlation (coefficient ≥0.5) with other variables. The validity of the model assumptions of linearity and homoscedasticity was assessed visually by plotting residuals against fitted values. The best-fitting model was selected by backwards selection starting from the full model and sequentially eliminating the term with the highest likelihood ratio test *p*-value until all terms in the model gave *p* ≤ 0.05. Analysis was performed in R programming language, version 4.2.1 (31). The coefficients and 95% CIs were exponentiated to give odds ratios representing the strength of association between each variable and the odds of a participant scoring a correct answer (which for the purpose of straightforward interpretation we refer to as knowledge level).

#### Assessing impact of engagement activities

The prevalence of negative perceptions of the impact of dog vaccination on dogs (such as dog will develop skin rashes, become infertile, docile or die when vaccinated), whether a participant had ever received training on dog behavior, dog handling and safe ways to interact with dogs, were quantified with proportions of “ yes/ no”. Participants also rated their abilities to communicate with dogs, restrain dogs, calm dogs down or hold dogs during vaccination: on a scale of “very low”, “low”, “average”, “high” and “very high”. Two-proportion Z-tests were used to compare responses before and after engagement to assess change in knowledge level and perceptions.

Mean scores on correct dog body language interpretation, dog handling, ways of avoiding dog attacks and ways of limiting injury when attacked for before and after engagement activities were compared with a Wilcoxon (Mann-Whitney U) test. Frequency charts were used to compare the reach of each medium delivered among follow up survey participants.

### Qualitative data

Audio recordings of FGDs were transcribed verbatim and translated from Kiswahili to English by a hired translator. The participant observation field notes and transcripts of the FGDs were inductively analyzed in NVivo 12 Plus version 20.5.1.940 (32). The files were read into NVivo and a thematic framework developed, which included the themes: perception of dogs and dog vaccination, bonding with dogs, dog body language interpretation (communication with dogs), facilitators and barriers to participation in dog vaccination, and dog restraining and handling. The thematic framework was applied in NVivo and the content of themes was then extracted into separate word files for referencing in presenting the results.

To demonstrate the feasibility of including communities in planning and implementing mass dog vaccination campaigns, meeting reports and the field observation notes on vaccination exercises were explored inductively using the thematic framework to confirm actual performance of assigned roles and to identify potential barriers and opportunities to community participation.

## Results

### Demographic information of participants of household survey

Table 1 summarizes information on demography and livelihoods of 728 respondents who took part in the household surveys across the two wards. Of these, 55% were female, most (64%) were between 20 and 49 years of age, they were predominantly farmers (85%), and about one-third (32%) did not have formal education. Only 31 respondents (4%) were unemployed; the main religions were Christianity (59%) and Islam (28%) and the majority (83%) were married. The two communities are broadly similar, except that mass vaccination campaigns have been happening in Kwihancha ward since Is 2003, and fishing communities are only present in Kyangasaga ward.

**Table 1:** Socio-demographic characteristics of household respondents.

**Variables**	**Categories**	**Ward**		**Totals (%)**
		**Kwihancha *n*(%)**	**Kyangasaga *n*(%)**	
Sex	Female	236 (32)	164 (23)	400 (55)
	Male	197 (27)	131 (18)	328 (45)
Age (years)	14-19	41 (6)	47 (6)	88 (12)
	20-49	269 (37)	195 (27)	461 (64)
	50>	123 (17)	53 (7)	176 (24)
Level of education	None	137 (19)	94 (13)	231 (32)
	Primary	275 (38)	186 (25)	461 (63)
	Secondary	21 (3)	15 (2)	36 (5)
Occupation	Unemployed	15 (2)	16 (2)	31 (4)
	Student	22 (3)	19 (3)	41 (6)
	Fishing	20 (3)	11 (2)	31 (5)
	Farmer	376 (51)	249 (34)	625 (85)
Religion	Traditional	9 (1)	12 (2)	21 (3)
	No Religion	41 (6)	28 (4)	69 (10)
	Islam	133 (18)	74 (10)	207 (28)
	Christian	250 (34)	181 (25)	431 (59)
Marital status	Single	54 (7)	54 (7)	108 (14)
	Married	362 (50)	236 (33)	597 (83)
	Widowed	17 (2)	5 (1)	22 (3)

### Population-related factors that predicted “knowledge” level of dog behavior, dog handling and safe interaction with dogs

Six out of 12 variables included in the model were significantly associated with knowledge level. The model predicted that: a 1 year increase in a participant's age, if a participant owned a dog, if a participant said he/she was taught how to hold a dog during vaccination and if a participant said he/she was threatened or bitten by dog were associated with 0.4%, 5%, 37% and 13% increased odds of scoring correct on knowledge level respectively. Whilst if a participant said he/she sent dog(s) for vaccination during the last vaccination campaign and if a participant said he/she was very afraid of dog (compared to those who said they were moderately afraid and not afraid) corresponded with 10% and 4% decreased odds of scoring correct on knowledge level respectively (Table 2).

**Table 2:** Participant-related factors that predicted 'knowledge level' of dog behavior and safe interaction with dogs.

**Variable**	**OR (95% CI)**	**Likelihood Ratio Tests**	
		**χ^2^**	***p*-value**
Intercept	7.5122 (5.8650–9.6222)	–	–
Age of participant	1.0040 (1.0021–1.0058)	17.512	0.0001
Participant owned a dog	1.0453 (1.0070–1.0851)	5.3892	0.0203
Trained on how to hold a dog	1.3695 (1.1341–1.6537)	10.755	0.0010
Ever bitten by a dog	1.1258 (1.0589–1.1969)	14.25	0.0002
Fear of dog	0.9595 (0.9315 – 0.9882)	7.5081	0.0061
Participated in last vaccination	0.9033 (0.8385–0.9733)	7.1079	0.0077
campaign			

### Comparison of participants' scores on dog body language interpretation, dog handling, ways of avoiding dog attacks and ways of limiting injury when attacked, before and after engagement activities

**Figure 2:**
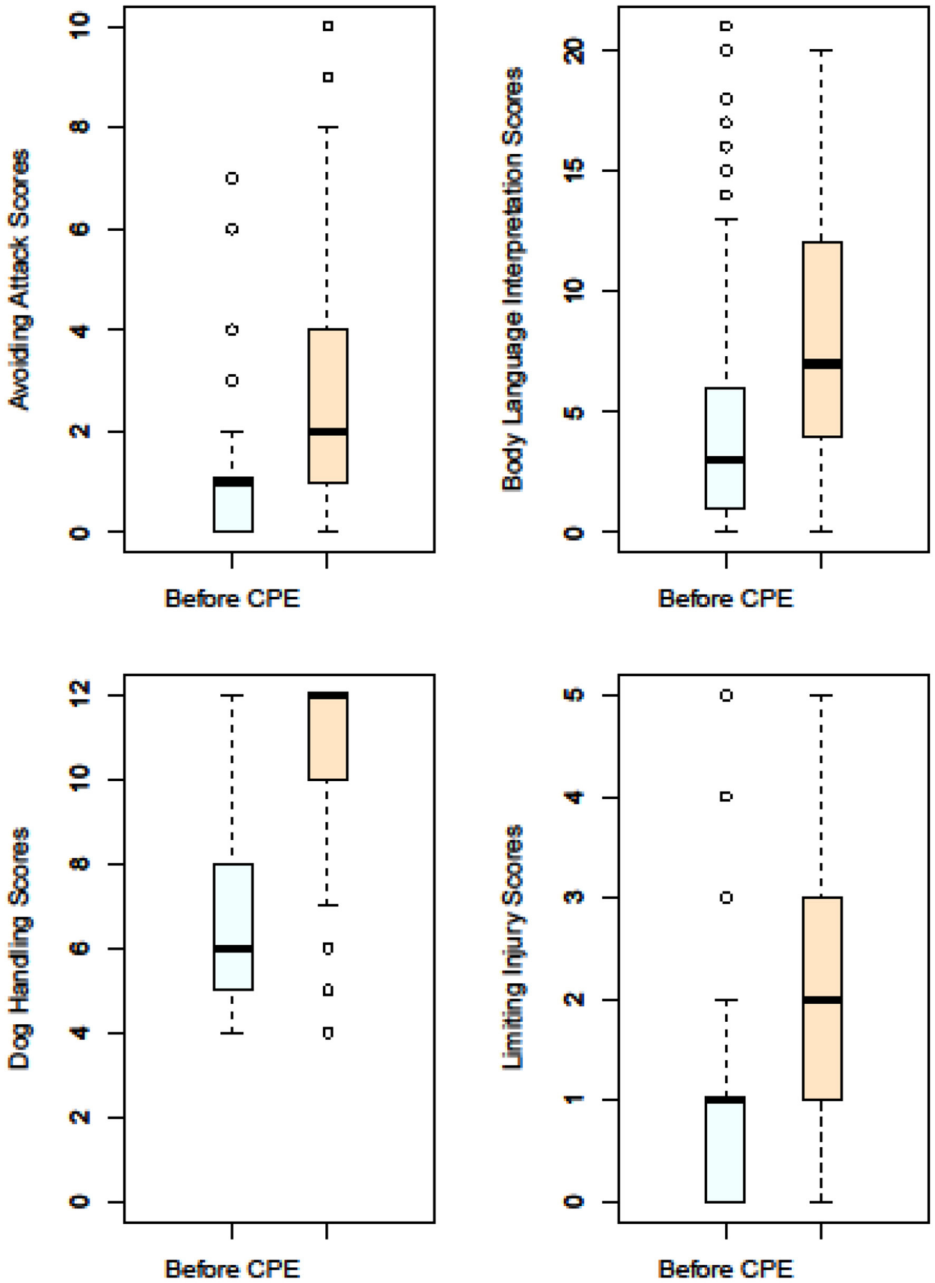
Scores on dog body language interpretation, ways of avoiding dog attacks, ways of limiting injury when attacked and dog handling, before and after engagement activities.

Mean scores on dog body language interpretation, dog handling, ways of avoiding dog attacks and ways of limiting injury when attacked were compared with a Wilcoxon M-W U test at *p* ≤ 0.05. All scores significantly improved after engagement activities (Figure 2). However, scores were low for both before and after. For example, 50% answered just three out of 21 questions correctly for interpretation of dog body language. The majority (86%) of participants responded that they were a little or very much afraid of dogs, but < half (41%) reported they had been bitten and/ or threatened by a dog.

### Participants' views on dog behavior, dog handling and safe interaction with dogs

The views of participants in FGDs suggested that dog behavior has implications for participation in dog vaccination as exemplified in the following quote. “*Another thing is the behavior of the dog, like being very reactive or calm and friendly. If a dog is very reactive, it barks at strangers and even attempts to chase them away. Therefore, that will be a major problem [for taking it to be vaccinated] and even if you take it to the vaccination center it will take a lot of strength to hold it because when it sees people different from those it sees at home it will react*” [A teenager, P3, FGD, Kwihancha].

In discussing barriers to sending dogs for vaccination, two themes dominated: distance to the vaccination point and relationship with the dog. “*[…]. If you are not used or close to your dog and just one day you want to tie it up with a rope or chain and take it for vaccination that will be a difficult case. […] because during the vaccination you have to hold your dog, if you can't hold it even the person who is giving the vaccination will be afraid because it is dangerous*” [An adult, P3, FGD, Kyangasaga].

Though knowledge of dog handling techniques was very low among study participants (none of the 16 dog owners and nine dog handlers demonstrated perfectly accurate knowledge as recommended), some had ideas of how to restrain a dog, making them confident in their ability to take their dogs for vaccination: “*I think nothing will make it difficult for me to take my dog for vaccination. If you see the dog showing signs that it does not want to go, you have to use other mechanisms since that is your dog and you know it very well. You can set a trap like a rope ready to catch it then you lure it with food, when it comes then you catch it and then you can take it for vaccination*” [An adult, P6, FGD, Kyangasaga].

### Perceptions of dog vaccination before and after engagement activities

Overall, only a few respondents (8%) held all four negative perceptions about the impact of dog vaccines at baseline, believing that vaccines will cause dogs: to die (5%), to develop skin rashes (1%), not reproduce (4%) and not to bark or hunt well (4%). The majority (92%) did not have any negative perceptions. Although the prevalence of negative perceptions was not significantly improved after engagement activities (Table 3), respondents' views from the review of the demonstration vaccination exercise suggested that participation in vaccination campaigns could help change negative views as this quote illustrates: “*after the vaccination they saw that their dogs did not die, they were happy and now they are asking when the dogs will be vaccinated again”* [Vaccinator, Demonstration Vaccination Review, Kyangasaga]. Many participants also expressed the view that vaccines provide protection: “*The vaccination is a kind of protection against diseases, that is to say, it is prior protection before a certain disease attacks the dog*” [A teenager, P3, FGD, Kwihancha], or does not have negative effects: “*Vaccination is a treatment for dogs, it eradicates the long-time diseases. So, if the dog gets vaccination, it will not affect it or make it unable to bark or to guard as usual, or not able to reproduce again. No, it will continue to do those things as usual and you have to train it*” [An adult, P6, FGD, Kwihancha].

**Table 3:** Change in perceptions of dog vaccination, interviewer and participant-rated knowledge of dog handling and safe interaction with dogs: before and after the engagement activity.

**Variables**	**Categories**	**Frequencies (%)**, ***n*** = **728**		**95% CI (difference: before-after)**	***p*-value**
		**Before**	**After**		
**Perceptions of dog vaccination**					
Vaccine causes rashes	No	687 (94)	714 (98)	−0.0416–0.0306	0.8163
Vaccine causes infertility	No	706 (97)	718 (99)	−0.0392–0.0337	0.9388
Vaccine reduces barking	No	708 (97)	722 (99)	−0.0392–0.0337	0.9388
Vaccine causes death	No	700 (96)	722 (99)	−0.0405–0.0322	0.8777
**Interviewer-rated knowledge of dog handling**					
Knowledge of ways of restraining dog	I don't know	124 (17)	13 (2)		
	Wrong	94 (13)	6 (1)		
	Partially correct	293 (40)	155 (21)		
	Correct	217 (30)	554 (76)	−0.0911−0.0353	0.0001
Knowledge of how to calm a dog	I don't know	222 (30)	18 (3)		
	Wrong	187 (26)	22 (3)		
	Partially correct	257 (35)	127 (17)		
	Correct	62 (9)	561 (77)	−0.1185−0.0683	0.0001
Knowledge of how to hold a small dog	I don't know	204 (28)	13 (2)		
	Wrong	255 (35)	27 (4)		
	Partially correct	205 (28)	104 (14)		
	Correct	64 (9)	584 (80)	−0.1230−0.0721	0.0001
Knowledge of how to hold a big dog	I don't know	203 (28)	11 (2)		
	Wrong	251 (34)	41 (6)		
	Partially correct	234 (32)	112 (15)		
	Correct	40 (6)	564 (77)	−0.1222−0.0729	0.0001
**Participants' self-rated knowledge of safe interaction with dog**					
Ability to communicate with a dog	Very low	252 (34)	7 (2)		
	Low	160 (22)	4 (1)		
	Average	224 (31)	122 (17)		
	High	85 (12)	515 (70)		
	Very high	7 (1)	80 (10)	−0.0226−0.0021	0.0155
Ability to restrain a dog	Very low	219 (30)	5 (1)		
	Low	133 (18)	8 (2)		
	Average	241 (33)	47 (6)		
	High	106 (15)	522 (71)		
	Very high	29 (4)	146 (20)	−0.0364−0.0076	0.0020
Ability to prevent dog attacks	Strongly disagree	42 (6)	1 (0)		
	Disagree	56 (8)	3 (0)		
	Can't tell	133 (18)	4 (1)		
	Agree	477 (65)	481 (66)		
	Strongly agree	20 (3)	239 (33)	−0.0584−0.0240	0.0001
Ability to limit injury when attacked by a dog	Strongly disagree	49 (7)	1 (0)		
	Disagree	74 (10)	2 (0)		
	Can't tell	222 (30)	10 (1)		
	Agree	370 (51)	610 (84)		
	Strongly agree	13 (2)	105 (15)	−0.0302−0.0055	0.0034

### Interviewer-rated and self-rated participants' knowledge of dog handling and safe interaction with dogs before and after engagement activities

Interviewer-rating of respondents' knowledge of ways of restraining and calming dogs at home and during vaccination clinics showed significant changes after engagement activities (Table 3 below).

Respondents' self-rating of their level of confidence in their abilities to communicate with their dogs, to restrain a dog and to avoid dog attacks or limit injury when attacked showed significant improvements after engagement activities (Table 3).

### Reach of intervention media among follow up survey participants

**Figure 3:**
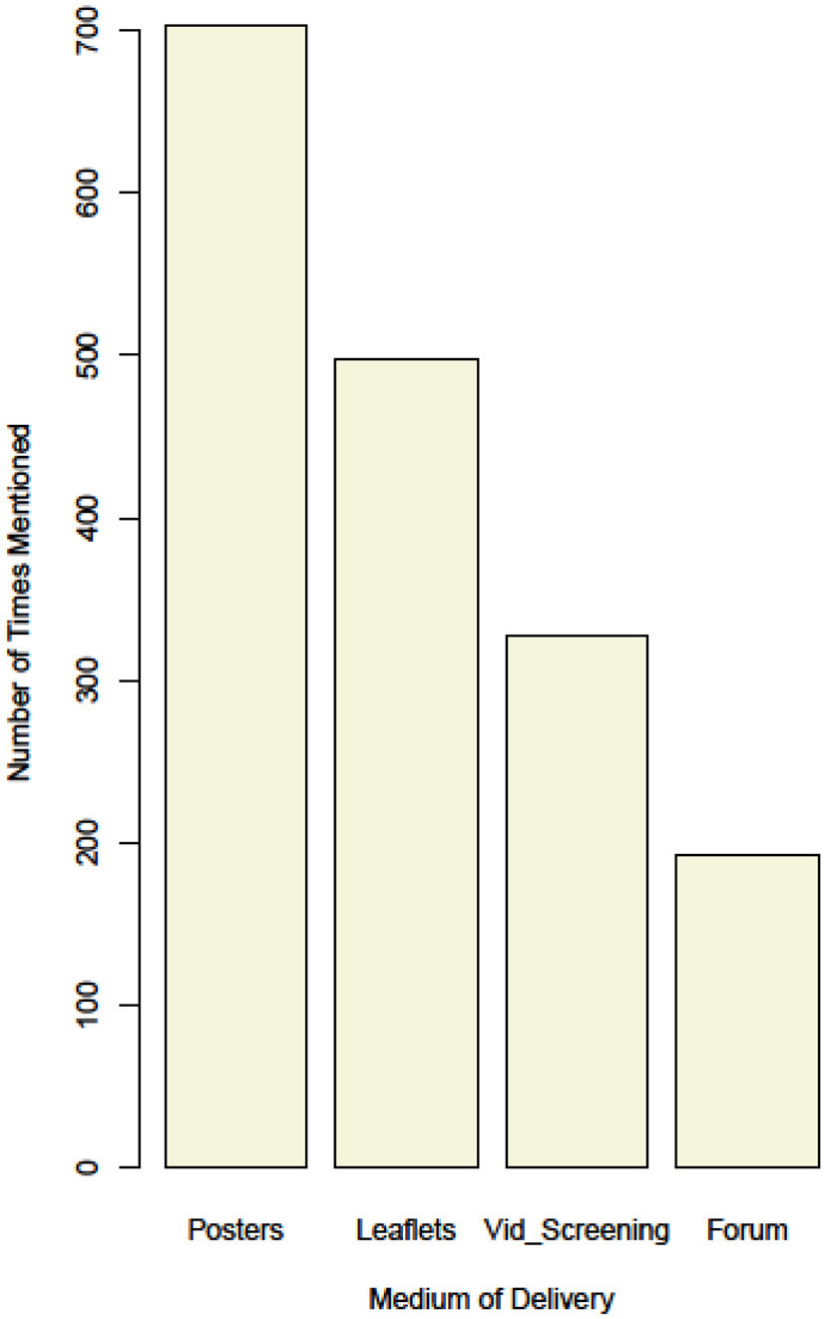
Reach of intervention media among follow up survey participants.

During the follow up survey, respondents indicated through which medium of delivery of engagement activity they received information on dog behavior and handling. Posters were the most frequently cited, followed by leaflets and village-level meetings (Figure 3). The distribution of posters and leaflets was targeted at participants in the survey, while village-level meetings and video screenings were targeted at the whole village. Respondents were further asked to indicate through which of the media they acquired new information the most; 81% referred to posters. During the follow-up survey, the majority of respondents were also observed to have the posters hanging on the wall in their sitting rooms.

### Feasibility of community participation in planning and implementing mass dog vaccination campaigns

To assess the feasibility of including community leadership in planning and implementing mass dog vaccination campaigns, an engagement meeting was held where the community engagement team, three district veterinary officers and 11 community leaders (one female), including the Ward Executive Officer, Village Executive Officers, Village and Subvillage Chairpersons participated. Their views on rabies in their communities were discussed and they identified what goes into organizing mass dog vaccination campaigns.

In all cases the district officers and community leaders agreed that rabies is a problem to their people and cited cases of dog bites and human deaths. The meeting then identified key mass dog vaccination activities and assigned them as roles to stakeholders (Table 4).

**Table 4:** Outcomes of meeting to involve community leadership in planning and implementing of dog vaccination: roles performed and those not performed.

**Stakeholder group**	**Assigned roles**	**Performed roles (yes/no)**
Research or donor organizations	Procure vaccine and vaccination materials (syringes, cards, register, transport, funds, megaphones)	Yes
	Recruitment and training of vaccinators	Yes
	Evaluation of outcome of vaccination clinics	Yes
Government or district veterinary office	Mobilizing stakeholders (researchers, donors, communities and ministry agencies) for sustained efforts	No
	Transport for vaccinators	No
	Provide cold boxes on the day of vaccination	Yes
	Issue directives in support of the vaccination campaign	Yes
	Ensure readiness of vaccinators	No
	Supervision of the vaccination clinics	No
Community leadership	Advertising of the campaign	Yes
	Provision of food for vaccinators in their village/sub-village during campaign	No
	Provision of waste bins	No
	Provision of table and chairs for vaccinators	Yes
	Conduct census of all dogs and cats per household	Yes
	Sub-village chairmen to assist the vaccinators at the vaccination points as dog handlers	Yes
	Conduct community self-assessment after the vaccination to see what proportion of dogs in each sub-village are vaccinated	No

Three months after the engagement meeting a vaccination campaign for the ward was undertaken to assess the feasibility of the stakeholders performing their respective roles. The community engagement team (representing research/ donor organizations) performed all of three assigned roles, the district veterinary office performed two out of six assigned roles and the community leadership performed four out seven assigned roles.

The district veterinary office cited lack of funds for supervision and to provide transportation for vaccinators. There was change in the community leadership of the ward between the engagement meeting and vaccination exercise, which affected performance of roles assigned to the community leaders (Table 4).

### Opportunities and barriers to community participation in planning and implementing mass dog vaccination campaigns

Our assessment of the meeting with the community leaders and observation of the demonstration vaccination exercise showed there were both opportunities and barriers to community participation in planning and implementing mass dog vaccination campaigns in the context of Tanzania. The opportunities included the availability of leaders at different levels (village, sub-village and ten-household units) of the community, and willingness of the community leaders to own and participate in planning and implementing of the dog vaccination campaign. Even though the community leaders agreed to assist the campaign for free, some demanded payments afterwards; one village executive officer said, “*for me I have my office to run and I have people I am working with and they have to be paid”* [Village Executive Officer, Field Note]. There also was a general lack of enthusiasm for dog vaccination, which hampered the mobilization for the vaccination campaign.

### Outcomes of the demonstration vaccination exercise

The census data showed a relatively low dog ownership: compared to other wards in Tanzania: total number of dogs for the ward was 566, ranged from 55 to 99 and averaged 81 dogs per sub-village. The vaccination coverage was good in most of the sub-villages: ranged 29-81% and averaged 59% (Table 5).

**Table 5:** Number of dogs censused and vaccinated in Kyangasaga Ward during the demonstration vaccination exercise.

**Villages**	**Sub-villages**	**Number of dogs**		**Vaccination coverage (%)**
		**Censused**	**Vaccinated**	
Gabimori	Ngurumi	95	60	63
	Mukiringo	60	42	70
	Buhare	73	59	81
	Esuka	86	53	62
Kyangasaga	Nsagaro	99	48	48
	Sonjo	55	16	29
	Kyangasaga	98	61	62

## Discussion

We assessed factors contributing to low owner participation in mass dog vaccination campaigns and whether they could be addressed through community engagement approaches, including how they impacted knowledge of dog behavior, handling, interactions, and perceptions of dog vaccination. The study also explored the feasibility of including community leaders in planning and implementing mass dog vaccination activities at community levels. Overall, we found that knowledge level of dog behavior, dog handling and safe interaction with dogs was associated to certain population-related factors including being older, owning a dog, receiving training on how to hold a dog, having been threatened or bitten by dog, having fear of dogs and participation in a recent vaccination campaign. Participants' knowledge level of how to restrain/ calm down a dog at home/ during vaccination, and participants' knowledge level of dog behavior and how to safely interact with a dog were low at the outset of the engagement activities but significantly improved after. The study recorded negative perceptions about the impact of dog vaccines on the dogs, though those were not widely held among the study population and were not significantly improved by the engagement activities. The engagement activities also demonstrated that communities can carry out simple activities and contribute to the processes of mass dog vaccination campaigns. The findings will guide planning of societal mobilization toward rabies elimination *via* mass dog vaccination.

It is widely documented that owner participation in dog vaccination campaigns is dependent upon their ability to restrain and take their dogs to the centers (7, 13–16). Hence it is interesting that participants' ability to interpret dog body language and their knowledge of techniques for restraining and holding dogs at home and during vaccination was very low among our study population. This phenomenon is also reported by studies conducted in the Flores Island of Indonesia, in Peru, in Tanzania and in Ethiopia, where the inability to restrain dogs was a common reason why owners failed to send their dogs for vaccination (7, 14, 16, 33). Also, poor knowledge of dog behavior and safe ways to interact with aggressive or stray dogs could be a precursor for dog bites, and could in turn discourage good relationships between people and dogs. Even though up to three-quarters of respondents at the outset of the study scored below average on knowledge of dog body language, knowledge of ways of averting dog attacks and of ways of limiting injury when attacked by a dog, scores on these variables were significantly improved after the engagement activities. This suggests that if the population is regularly engaged and provided with information on these topics, knowledge will improve and potentially the ability to take dogs for vaccination. This is in line with findings from a study on what determines intention of owners to participate in dog vaccination campaigns (34).

It is also noteworthy that participants' baseline knowledge of dog behavior and handling, and safe ways to interact with dogs, was not significantly associated with level of education. In a rural setting in Tanzania, older residents are less likely to have a high level of education (32% had no formal education): but as shown by the model, older people will tend to have higher knowledge levels. Similarly, is interesting that participating in a recent vaccination campaign was negatively associated with knowledge level. This could be explained by the observation that knowledge level increased with age but it is young people who usually take the households' dog(s) for vaccination (25, 26). Understanding population-related factors that predicted knowledge level on dog behavior, dog handling and safe ways to interact with dogs can be useful in defining the target group for interventions aimed at mobilizing the population for mass dog vaccination campaigns. An individual's knowledge on dog behavior and handling techniques has implications for the frequency of dog bites, willingness of people to be close to their dogs to give them the care that they need and hence participate in mass dog vaccination. These have been cited as barriers to owner participation in mass dog vaccination campaigns in Peru, the Philippines, Indonesia and Grenada (7, 13–15).

In this regard, opportunities exist at village level for regular delivery of talks on dog behavior and dog vaccination to communities. This can either be assigned to ward-level livestock field officers (in the context of Tanzania) as part of their animal health extension duties or to duly selected community-based people with some knowledge of animal husbandry practices who can be trained to deliver these talks at community meetings. These lay people have previously been used in Tanzania to deliver Newcastle Disease (35–37) and rabies (10) vaccinations to animals. Similarly, community structures or people also have been used in communicating programme objectives and benefits to communities (37). This study showed that people are likely to benefit from information prepared in the forms of posters and leaflets which they potentially could keep for long periods. The majority of respondents in this study were found to still have the posters neatly pasted in their living rooms 3 months after they were given out.

Regularly engaging community members with information on dogs and rabies could also help correct negative perceptions such as dogs will develop skin rashes, become infertile, docile or die when vaccinated (16). Although these negative perceptions were not widely held among our study population, they could have significant influence on owner participation if held by a socially important figure. The negative perceptions not being significantly influenced by the engagement activities could be due to the fact that these views were held by very few people and for a very long time, and could not be improved through engagement only. It might take people actually vaccinating their dogs and observing the outcomes in order to change their perceptions, as owners' intention to vaccinate their dogs was found to be positively associated with perceived benefits and trust in the vaccine (16). However, it would be useful to systematically investigate and document adverse events after dog vaccination to inform community engagements toward dog vaccination. For instance, people may associate the high mortality rates in puppies with vaccination and could reinforce belief that the vaccine causes death. It is possible that some forms of adverse events occur on a small scale in vaccinated dogs but these may be insignificant compared to the benefits of the vaccine. A study conducted in the Philippines actually reported that owners of 20% of vaccinated dogs said they observed some form of adverse reactions in their pets (13). The limited prevalence of these perceptions also suggests the need to focus on other known barriers to owner participation such as ability to restrain dogs and charging of fees for vaccination.

Sustaining the interest of community leaders in discussing dog vaccination can be challenging. In the fishing communities of this study for example, dogs are usually seen as a nuisance by non-dog owners because they eat their sardines (dried in the open). There also is the impression from livestock keepers that getting treatment for livestock diseases is of higher priority than dog vaccination. Integrating approaches for local disease control programmes could help foster interest. For instance, discussing dog vaccination alongside vaccination for other livestock, enforcement of local dog vaccination laws and recognition or rewarding of community leaders where high vaccination coverage is achieved could foster prioritization of dog vaccination. Also, media platforms such as national television and radio stations can deliver regular segments as part of national mass dog vaccination mobilization strategies to inform the population on dog behavior and dog handling techniques. The means of communication (posters, leaflets, flip charts, video screenings and village level forums) used in the context of this engagement were extensive and intensive, and likely explain why they had significant impact on knowledge. Therefore, it will require much commitment to scale up and sustain these means of engagement at national levels due to cost. The pictorial illustrations of posters also may have aided learning even by those who could not read.

Outlining activity components of mass dog vaccination campaigns and assigning roles to communities with participation of community leaders showed it is feasible for communities to participate fully in the planning and execution of mass dog vaccination campaigns. This can be by contributing both simple material and human resources with the potential to reduce campaign costs. For instance, community-based people were involved in the development of a low-tech, passive cooling clay device for storing canine rabies vaccines in villages in Tanzania (38). Arguably, communities can contribute locally made waste bins, in addition to tables and chairs, registers, and advertising of campaigns (39). This was also demonstrated by mass dog vaccination campaign cost components description studies in Chad and Kenya (21–23). A review also found that community participation in planning and delivery of interventions was the most frequently cited facilitating factor in the success of the community-based, lay animal vaccination programmes (40). However, a larger study is needed to establish the feasibility and sustainability of community participation in mass dog vaccination.

The community leaders actually performed most of the roles assigned to them during the demonstration vaccination in this engagement, lending credence to the feasibility of their participation. Another example of local contribution is community self-monitoring of locally delivered intervention and is shown to be effective at promoting reach and sustainability (19, 41) and can be employed in mass dog vaccination campaigns. While the community structure of Tanzania is suitable for inclusion in planning, organizing and monitoring of dog vaccination campaigns, certain barriers such as lack of traction for volunteerism and lack of prioritization of welfare of dogs exist and may hamper strong representation of dog vaccination on the agenda of communities. For example, community people asking for incentives was cited to have derailed implementation of community self-monitoring in the community-directed treatment with ivermectin”programme (29). However, these barriers could be removed with comprehensive community entry processes or with incentives (such as enlisting the organization of mass dog vaccination as part of the job description for community leaders and linking of rabies outcomes to their promotion) to fully bring communities on board with mass dog vaccination campaigns. Again, deepening consensus on community roles and possibly crafting local government legislations or by-laws to back them can be helpful.

The overall coverage of the demonstration vaccination was a little above average. Given the low dog population per sub-village, it can be expected that almost all of the dogs should have been vaccinated. However, the mobilization likely was affected by the fact that these communities were new to mass dog vaccination, the mobilization being volunteer-based and also there was a change in the community leadership between the time of planning and execution of the vaccination exercise. The vaccination exercises were conducted just few weeks after Covid-19 related restrictions on social gathering was removed in Tanzania.

The key strength of this study is that it compared participants who received the full range of engagement activities before and after, using the same sets of questions. However, the field data collectors who evaluated responses after engagement were not blinded.

## Conclusion

This study found low level of knowledge on effective and safe dog handling techniques among the population, which could make people uncertain in their ability to restrain and take their dogs to vaccination centers. Interacting with communities on rabies and dog vaccination improved their knowledge of dog behavior and handling and their perceptions of the benefits of dog vaccination. Results also showed community members can deliver components of mass dog vaccination campaigns, including planning and delivery processes, with potential for cost savings.

## Data availability statement

The raw data supporting the conclusions of this article will be made available by the authors, without undue reservation.

## Ethics statement

The study was approved by the Institutional Animal Care and Use Committee, Washington State University [Approval No. 04577 – 001], the Tanzania National Medical Research Institute [NIMR/HQ/R.8a/Vol.IX/2788] and the Ifakara Health Institute [IHI/IRB/No:024-2018]. Administrative permissions were from Rorya and Tarime veterinary offices and leaderships of Kwihancha and Kyangasaga wards. Written informed consent to participate in this study was provided by the participants' legal guardian/next of kin.

## Author contributions

CTD and KK conceptualize the community engagement. CTD, KK, SC, KH, FL, and SW participated in the development of the study methodology and coordination of the field activities. CTD conducted the engagement, data collection activities with support of field data collectors and produced the first draft of the manuscript. CTD, KK, EF, and PJ performed the analysis and interpretation of data, defined outline of the manuscript. All authors participated in review and editing of the manuscript. All authors contributed to the article and approved the submitted version.

## Funding

Funding for this work was provided by the African Academy of Sciences (AAS) [CPE/19//082]. CTD, SC, and KK: CTD received a PhD fellowship from the DELTAS Africa Initiative [Afrique One-ASPIRE /DEL-15-008]. Afrique OneASPIRE is funded by a consortium of donors, including the African Academy of Sciences (AAS), Alliance for Accelerating Excellence in Science in Africa (AESA), the New Partnership for Africas Development Planning and Coordinating (NEPAD) Agency, the Wellcome Trust [107753/A/15/Z] and the UK government. Funding support was provided by the National Institutes of Health [R01AI141712] (to FL, SC, KH, and SW). The content is solely the responsibility of the authors and does not necessarily represent the official views of the National Institutes of Health. Funding was provided by Wellcome [207569/Z/17/Z] (KH and CTD] and and MSD Animal Health (FL and KH).

## Conflict of interest

The authors declare that the research was conducted in the absence of any commercial or financial relationships that could be construed as a potential conflict of interest.

## Publisher's note

All claims expressed in this article are solely those of the authors and do not necessarily represent those of their affiliated organizations, or those of the publisher, the editors and the reviewers. Any product that may be evaluated in this article, or claim that may be made by its manufacturer, is not guaranteed or endorsed by the publisher.
