# Supplementary material for: Participation in mass dog vaccination campaigns in Tanzania: Benefits of community engagement
Source: Front Public Health. 2022 Oct 13;10:971967. doi: 10.3389/fpubh.2022.971967 (PMC9616113; doi:10.3389/fpubh.2022.971967)
Supplement: Supplementary file 1 [file Data_Sheet_1.docx]

**SUPPLEMENTARY FILES**

**File 1: Baseline Household Survey Questionnaire**

**SECTION A: PARTICIPANT DEMOGRAPHIC INFORMATION**

| **District: _______________ Ward:_______________ Village:_______________ Sub-Village: _________**  **Interviewer ID : _________ Interview Date: dd/mm/yy: …..../03/2020 Household GPS: _________** |
| --- |

01. Individual ID: ……………..

02. Gender: Male [ ] Female [ ]

03. Age: …….…….

04. What is the highest level of education you have attained?

None [ ] Primary [ ] Voc Training or Skilled [ ] O’ Level [ ] A’ Level [ ] Tertiary [ ]

05. What is your occupation?

Unemployed [ ] Farming or hunting [ ] Trading [ ] Salaried worker [ ] Artisan [ ] Student [ ] Other (Specify) ………………………………..… …

06. What is your religion? Catholic [ ] Lutheran [ ] Pentecostal [ ]

Islam [ ] Traditionalist [ ] No religion [ ] Other (Specify)…………..……………

07. What is your marital status now?

Single [ ] Married [ ] Widowed [ ] Divorced [ ] Separated [ ]

**SECTION B: INTRODUCTION**

08. Do you have a dog? YES □ NO MORE □ NEVER □ I WILL LIKE TO □

09. If yes, for how long? ………………..

**SECTION C: DOG BEHAVIOR**

10. Have you ever received any education on dog behavior? YES □ NO □

11. If yes, how would rate your knowledge on dog behavior?

Very High □ High □ Average □ Low □ Very Low □

12. I can easily communicate with my dog(s) and it/they obey me

Strongly agree □ Agree □ Can’t tell □ Disagree □ Strongly disagree □

13. I can easily recognize when my dog is communicating something to me

Strongly agree □ Agree □ Can’t tell □ Disagree □ Strongly disagree □

14. What does it mean when your dog shows the following behaviors/body languages/sounds? *(Read out each behavior to participant. Show picture where participant can’t recognize your description), to be scored.*

□Dog licks the mouth

□Dog licks nose

□Dog ears stand up with eye widely opened and neck raised or stretched forward

□Dog moves back whiles barking or not barking

□Dog looks away with head turned and walks away

□Dog lies back down and rubs belly and nose

□Dog stretches and touches person friendlily

□Dog tilts head and looks at you

□Dog paces around

□Dog show wide eyes with white portion becoming more visible

□Dog digs

□Dog wags tail

□Tail tugged between legs

□Tail standing vertically upwards

□Tail pointing forward with hair on body standing

□Dog sits and raises one hand towards a person

□Dog Barks

□Dog Wheezes

□Dog Howls

□Dog Growls

□Dog Snarls

**SECTION D: DOG CATCHING & HANDLING**

15. Have you ever received any training on how to catch your dog at home?

YES □ NO □

16. If yes, how would you rate your ability to catch your dog at home?

Very High □ High □ Average □ Low □ Very Low □

17. I can comfortably send my dog for vaccination without using force

Strongly agree □ Agree □ Can’t tell □ Disagree □ Strongly disagree □

18. Tell me the effective ways to catch your dog

Correct □ Somehow □ Wrong □

19. Have you ever received any training on how to hold your dog for it to be vaccinated?

YES □ NO □

20. I can comfortably hold my dog for it to be vaccinated

Strongly agree □ Agree □ Can’t tell □ Disagree □ Strongly disagree □

21. How do you calm your dog down to be vaccinated?

Correct □ Somehow □ Wrong □

22. Describe the correct ways to hold a small dog for it be vaccinated?

Correct □ Somehow □ Wrong □

23. Describe the correct ways to hold a big dog for it be vaccinated?

Correct □ Somehow □ Wrong □

**SECTION E: POSITIVE INTERACTION WITH DOG**

24. Has a dog ever attempted to bite you or someone you know? YES □ NO □

Are you afraid of dog? VERY MUCH □ MUCH □ I DON’T NOW □ A LITTLE □ NOT AT ALL □

25. When I meet a strange dog in its home or outside, I know how to react to avoid the dog attacking me. Strongly agree □ Agree □ Can’t tell □ Disagree □ Strongly disagree □

26. Tell me how you will act to avoid attack? *Check those mentioned by participant.*

□Avoid eye contact with dog

□Turn your body slowly to the side

□Cross your hands

□Completely ignore the dog

□Be still for a short period then move slowly away

□Do not show bad actions towards dog such hitting it with stone

□Avoid shouting, noise irritates the dog or can read your mood from your voice

□Use nice/low voice and snapping of the fingers to show love to dog

□Calmly ask someone nearby to help or

□Stand with confidence until someone/owner comes or dogs finishes inspecting you

□If you are in the ground slowly curl into a ball on your knees with your hands clasped behind your neck protecting your head and throat

27. If the strange dog decides to attack me, I know how to defend myself to avoid or limit injury

Strongly agree □ Agree □ Can’t tell □ Disagree □ Strongly disagree □

28. Tell me how you will prevent or limit injury when the dog attacks? *Check those mentioned by participant.*

□Swerve if you can

□Find a barrier that you can put between you and the dog – a purse, rolled-up jacket or a stick and try to redirect the dog’s bites onto that item.

□Find higher ground and try to move to a position of height away from the dog. It is much harder for him to bite effectively from below.

□Try not to scream as this could antagonize the dog further.

□Cover the dog’s head with a shirt or blanket so that he cannot see. If you block his eyesight for a moment, it might provide you a window to escape.

**SECTION F: PERCEPTION OF DOG VACCINES**

29. Do you think vaccinating your dog can cause your dog to develop skin rashes? YES □ NO □

30. Do you think vaccinating your dog can cause your dog not to reproduce well? YES □ NO □

31. Do you think vaccinating your dog can cause your dog not to bark at night? YES □ NO □

32. Do you think vaccinating your dog can cause your dog to die? YES □ NO □

33. Did you send your dog for vaccination the last time dogs were vaccinated here? YES □ NO □

**File 2: Follow up Household Survey Questionnaire**

**SECTION A: PARTICIPANT DEMOGRAPHIC INFORMATION**

| **District: _______________ Ward:_______________ Village:_______________ Sub-Village: ________**  **Interviewer ID : _________ Interview Date: dd/mm/yy: …..../03/2020 Household GPS: _________** |
| --- |

1. Individual ID: ……………..

**SECTION C: DOG BEHAVIOR**

2. Through which of the following means did you receive education from us n dog behavior, how to catch your dog at home and hold it during vaccination?

Village level teaching □ Cinema □ Posters □ Leaflets □ None of them □

3. Through which of them did you learn more?

Village level teaching □ Cinema □ Posters □ Leaflets □ None of them □

4. If yes, how would rate your knowledge on dog behavior NOW?

Very High □ High □ Average □ Low □ Very Low □

5. You can NOW easily communicate with your dog(s) and it/they obey you, right?

Strongly agree □ Agree □ Can’t tell □ Disagree □ Strongly disagree □

6. You can NOW easily recognize when my dog is communicating something to me

Strongly agree □ Agree □ Can’t tell □ Disagree □ Strongly disagree □

7. What does it mean when your dog shows the following behaviors/body languages/sounds? *(Read out each behavior to participant. Show picture where participant can’t recognize your description), to be scored.*

□Dog licks the mouth

□Dog licks nose

□Dog ears stand up with eye widely opened and neck raised or stretched forward

□Dog moves back whiles barking or not barking

□Dog looks away with head turned and walks away

□Dog lies back down and rubs belly and nose

□Dog stretches and touches person friendlily

□Dog tilts head and looks at you

□Dog paces around

□Dog show wide eyes with white portion becoming more visible

□Dog digs

□Dog wags tail

□Tail tugged between legs

□Tail standing vertically upwards

□Tail pointing forward with hair on body standing

□Dog sits and raises one hand towards a person

□Dog Barks

□Dog Wheezes

□Dog Howls

□Dog Growls

□Dog Snarls

**SECTION D: DOG CATCHING & HANDLING**

8. How would you rate your ability to catch your dog at home NOW?

Very High □ High □ Average □ Low □ Very Low □

9. You can NOW comfortably send my dog for vaccination without using force

Strongly agree □ Agree □ Can’t tell □ Disagree □ Strongly disagree □

10. Tell me the effective ways to catch your dog

Correct □ Somehow □ Wrong □

11. You can NOW comfortably hold my dog for it to be vaccinated

Strongly agree □ Agree □ Can’t tell □ Disagree □ Strongly disagree □

12. How do you calm your dog down to be vaccinated?

Correct □ Somehow □ Wrong □

13. Describe the correct ways to hold a small dog for it be vaccinated?

Correct □ Somehow □ Wrong □

14. Describe the correct ways to hold a big dog for it be vaccinated?

Correct □ Somehow □ Wrong □

**SECTION E: POSITIVE INTERACTION WITH DOG**

15. When you meet a strange dog in its home or outside NOW, you know how to react to avoid the dog attacking you.

Strongly agree □ Agree □ Can’t tell □ Disagree □ Strongly disagree □

16. Tell me how you will act to avoid attack? *Check those mentioned by participant.*

□Avoid eye contact with dog

□Turn your body slowly to the side

□Cross your hands

□Completely ignore the dog

□Be still for a short period then move slowly away

□Do not show bad actions towards dog such hitting it with stone

□Avoid shouting, noise irritates the dog or can read your mood from your voice

□Use nice/low voice and snapping of the fingers to show love to dog

□Calmly ask someone nearby to help or

□Stand with confidence until someone/owner comes or dogs finishes inspecting you

□If you are in the ground slowly curl into a ball on your knees with your hands clasped behind your neck protecting your head and throat

17. NOW if a strange dog decides to attack you, you know how to defend myself to avoid or limit injury

Strongly agree □ Agree □ Can’t tell □ Disagree □ Strongly disagree □

18. Tell me how you will prevent or limit injury when a dog attacks you? *Check those mentioned by participant.*

□Swerve if you can

□Find a barrier that you can put between you and the dog – a purse, rolled-up jacket or a stick and try to redirect the dog’s bites onto that item.

□Find higher ground and try to move to a position of height away from the dog. It is much harder for him to bite effectively from below.

□Try not to scream as this could antagonize the dog further.

□Cover the dog’s head with a shirt or blanket so that he cannot see. If you block his eyesight for a moment, it might provide you a window to escape.

**SECTION F: PERCEPTION OF DOG VACCINES**

19. Do you think vaccinating your dog can cause your dog to develop skin rashes? YES □ NO □

20. Do you think vaccinating your dog can cause your dog not to reproduce well? YES □ NO □

21. Do you think vaccinating your dog can cause your dog not to bark at night? YES □ NO □

22. Do you think vaccinating your dog can cause your dog to die? YES □ NO □

23. Did you send your dog for vaccination the last time dogs were vaccinated here? YES □ NO □

**File 3: Topic guides for focus group discussions**

**Introduction**

Tell me what you think about dogs as friends and help to humans.

What are the ways you or other people you know use dogs?

1. What make you like or dislike to be close to dogs?

2. How do you build good relationship with your dog?

3. Have you trained your dog? Describe the methods you used to train your dogs.

4. Describe the ways you use to communicate to your dog to obey you and how it communicates back to you.

5. How can you tell if a dog is dangerous and what will you do in such a situation?

6. What do you think of dog vaccination?

7. What do you think will make it easy or difficult for you to take your dog to vaccination?

**File 4: The CPE interventions, number of people reached and how they were evaluated**

| **Intervention** | **Number of people reached** | | **Duration of delivery** | **How outcomes were evaluated** |
| --- | --- | --- | --- | --- |
|  | **Kwihancha** | **Kyangasaga** |  |  |
| Information via posters illustrations and leaflets | 472 | 354 | Distributed at baseline, found pasted in sitting rooms at follow (3 months later) | Measurement of knowledge gained through questionnaires |
| Sub-village-level (7) and school-based (3) fora (conversations) with flipcharts and videos | 1,062 | 950 | 7 days, 2 hours for a forum and 2 hours for video screening, separately in afternoon and evening respectively. | Meetings reports and observation field notes |
| Dog handling demonstration by dog owners and vaccinators | 8 CPE team members  9 dog handlers during vaccination | 8 CPE team members  16 dog owners | 4 days, 3-4 hours each | Observation field notes |
| Mass dog vaccination planning meeting with community leaders and district veterinary officials | 8 CPE team members  11 community leaders | 8 CPE team members  3 district veterinary officials  13 community leaders | 2 days, 3 hours 45mins each | Meetings reports, observation field notes and documenting participation in a vaccination exercise |

**File 5a: Pictorial aid for recommended dog body language interpretation**


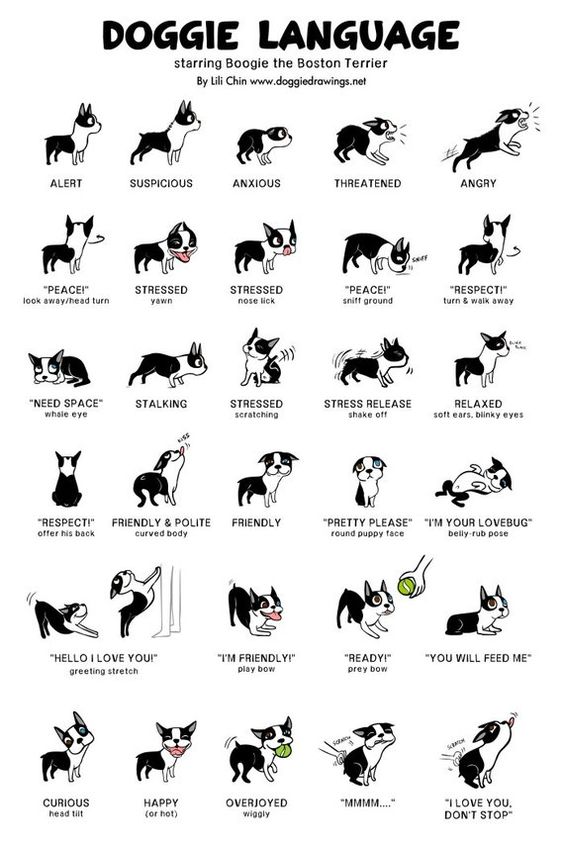


Available at: <https://bowwowinsurance.com.au/pet-community/pet-talk/dog-communication-body-language/>

**File 5b: Posters for short-medium term learning about recommended ways to avoid being attacked and limiting injury when attacked (a-b); recommended ways to hold dog during vaccination (c) and uses of dogs (d).**


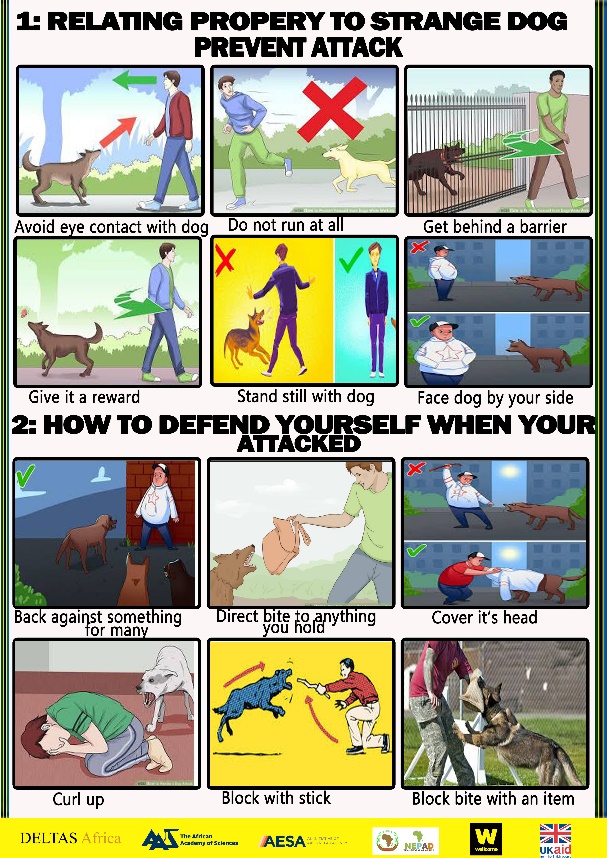

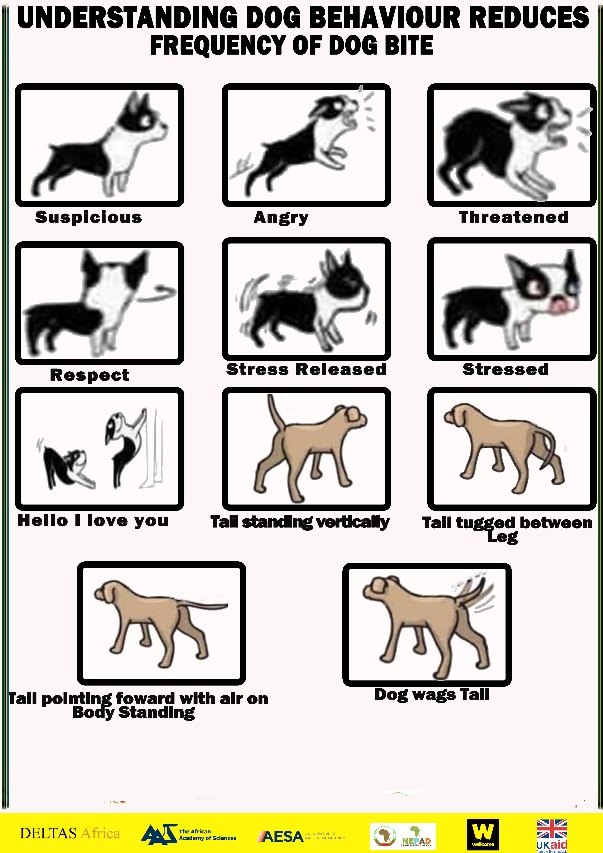


Avoid being attacked and limiting injury when attacked Available at: <https://www.google.com/search?q=avoiding+attack+by+dogs&tbm=isch&ved=2ahUKEwiI76ac2t73AhX8QfEDHY5rCVgQ2-cCegQIABAA&oq=avoiding+attack+by+dogs&gs_lcp=CgNpbWcQAzoHCAAQsQMQQzoECAAQQzoFCAAQgAQ6BggAEAgQHlDyHFiRcWC2dWgAcAB4AIABsAGIAaIVkgEEMjkuNJgBAKABAaoBC2d3cy13aXotaW1nwAEB&sclient=img&ei=A3p_YsjhJfyDxc8PjtelwAU#imgrc=7rTV7rzz5IJiKM>


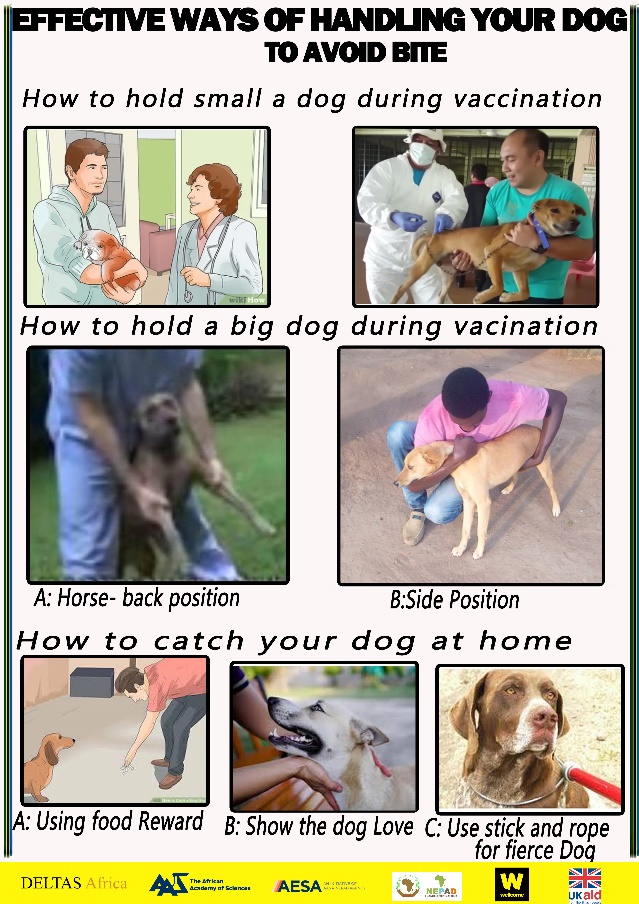

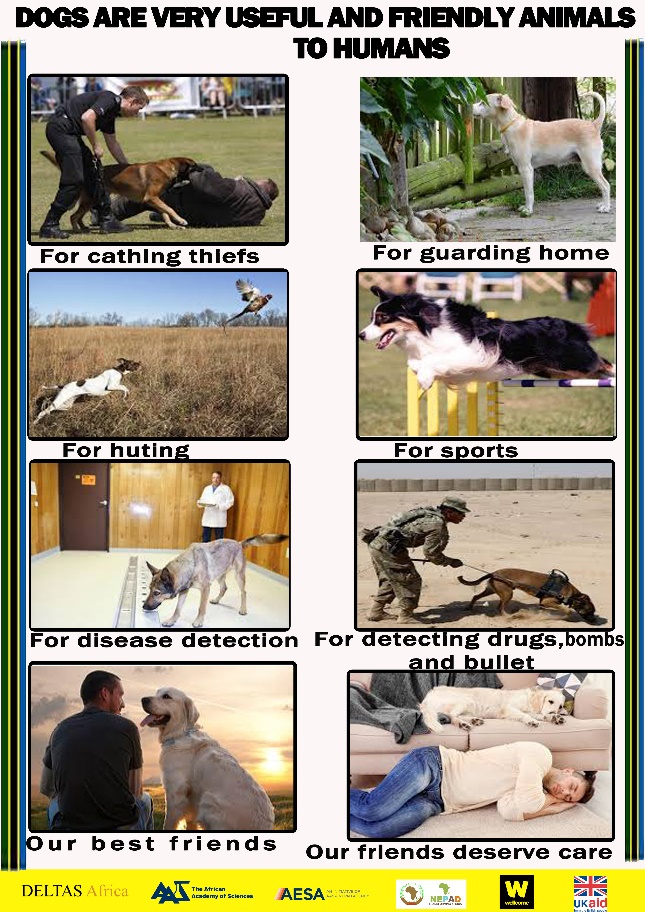


Recommended ways of holding dogs during vaccination, available at: <https://www.google.com/search?q=how+to+hold+dog+during+vacciantion&tbm=isch&ved=2ahUKEwj87OH1_d73AhXI0oUKHd6OAEQQ2-cCegQIABAA&oq=how+to+hold+dog+during+vacciantion&gs_lcp=CgNpbWcQAzoECCMQJzoECAAQQzoFCAAQgAQ6CwgAEIAEELEDEIMBOggIABCABBCxAzoICAAQsQMQgwE6BwgAELEDEEM6BAgAEB46BggAEAgQHjoECAAQGFCGK1jqnAFggJ8BaAFwAHgAgAGiAYgBwReSAQQzMC42mAEAoAEBqgELZ3dzLXdpei1pbWfAAQE&sclient=img&ei=cp9_YvzrHMillwTenYKgBA>

Common uses of dogs, available at: <https://www.google.com/search?q=uses+of+dogs&tbm=isch&ved=2ahUKEwi-xp6A_t73AhUC04UKHd1pBWsQ2-cCegQIABAA&oq=uses+of+dogs&gs_lcp=CgNpbWcQAzIFCAAQgAQyBQgAEIAEMgQIABAYMgQIABAYMgQIABAYMgQIABAYMgQIABAYOgQIIxAnOggIABCABBCxAzoLCAAQgAQQsQMQgwE6BAgAEEM6CggAELEDEIMBEEM6CAgAELEDEIMBUNmmBliAugZglr8GaABwAHgAgAFxiAG6CJIBBDEzLjGYAQCgAQGqAQtnd3Mtd2l6LWltZ8ABAQ&sclient=img&ei=iJ9_Yr7iGoKmlwTd05XYBg>

**File 5c: Leaflets for short-medium term learning about dogs and rabies**


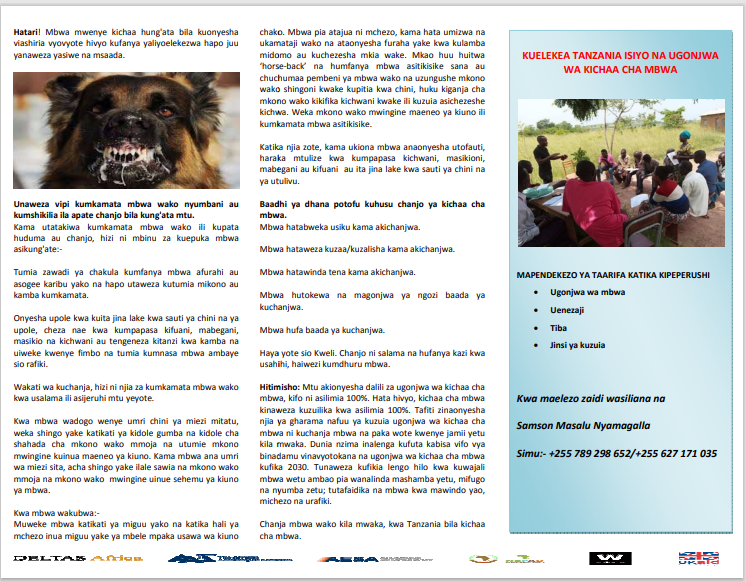

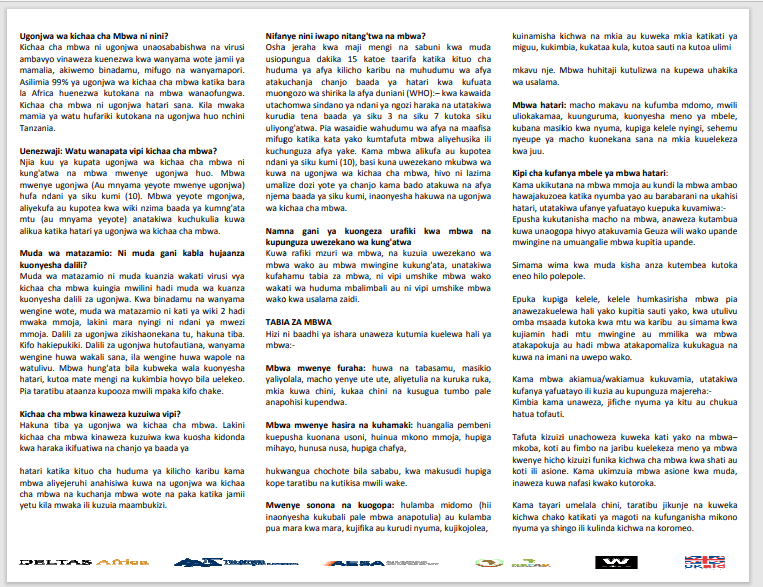


**File 5d: Links to videos:**

Recommending ways to void dog attack, available at: <https://www.youtube.com/watch?v=OrkvjdlWWG0>

Recommending ways to survive dog attacks, available at: <https://www.youtube.com/watch?v=kaA2urO2vCA> and <https://www.youtube.com/watch?v=vX-OOfbnD9w>

Recommended ways to restrain a dog during vaccination, available at: <https://www.youtube.com/watch?v=ULFwjoZoRt8>

**File 5e: International guides**

We referred to the International Companion Animal Management Coalition guide, available at: <https://www.icam-coalition.org/wp-content/uploads/2019/09/2019-ICAM-DPM-guidance-Interactive-updated-15-Oct-2019.pdf>

And World Society for the Protection of Animals, available at: <https://www.icam-coalition.org/wp-content/uploads/2017/03/Identification-methods-for-dogs-and-cats.pdf>

**File 6: Proforma for observing community leadership involvement in the vaccination exercise**

| **TOPICS** | **NAMES OF SUB-VILLAGES** | | | | | | | |
| --- | --- | --- | --- | --- | --- | --- | --- | --- |
|  | Esuka | Mkiringo | Buhare | Sonjo | Kyangasaga | Esegere | Ngurumi | Nyasagaro |
| Set up and commencement of vaccination |  |  |  |  |  |  |  |  |
| Sub-village chairs went round houses to conduct census of all dogs and cats per households? |  |  |  |  |  |  |  |  |
| Advertising of the campaign |  |  |  |  |  |  |  |  |
| Provision of table and chairs for vaccinators |  |  |  |  |  |  |  |  |
| Provision of waste bins |  |  |  |  |  |  |  |  |
| Provision of meals to vaccinators or the respective leaders should cook for vaccinators working in their village/sub-village during campaign |  |  |  |  |  |  |  |  |
| Sub-village chairmen to assist the vaccinators at the vaccination points as dog handlers |  |  |  |  |  |  |  |  |
| Conduct community self-assessment after the vaccination to see what proportion of dogs in each sub-village are vaccinated |  |  |  |  |  |  |  |  |
| General observation of:  -People’s attitudes  -Turn outs  -Events |  |  |  |  |  |  |  |  |
